# Probing Nucleic Acid Interactions and Pre-mRNA Splicing by Förster Resonance Energy Transfer (FRET) Microscopy

**DOI:** 10.3390/ijms131114929

**Published:** 2012-11-14

**Authors:** Eva Šimková, David Staněk

**Affiliations:** Institute of Molecular Genetics, Academy of Sciences of the Czech Republic, Videnska 1083, 142 20 Prague, Czech Republic; E-Mail: eva.simkova@img.cas.cz

**Keywords:** FRET, FLIM, acceptor photobleaching, RNA interactions, spliceosome

## Abstract

Förster resonance energy transfer (FRET) microscopy is a powerful technique routinely used to monitor interactions between biomolecules. Here, we focus on the techniques that are used for investigating the structure and interactions of nucleic acids (NAs). We present a brief overview of the most commonly used FRET microscopy techniques, their advantages and drawbacks. We list experimental approaches recently used for either *in vitro* or *in vivo* studies. Next, we summarize how FRET contributed to the understanding of pre-mRNA splicing and spliceosome assembly.

## 1. Introduction

Förster resonance energy transfer (FRET) is a photophysical phenomenon that was first described by Theodor Förster in the 1940s [1]. FRET is a non-radiative energy transfer from the excited donor fluorophore to a nearby acceptor fluorophore, which is mediated by dipole-dipole interactions. Many different fluorophores can be used as FRET donors or acceptors [2]. Those most commonly used in cell biology are either small fluorescent molecules which can be utilized for covalent labeling [3] or genetically encoded tags, such as fluorescent proteins [4].

**Figure 1:**
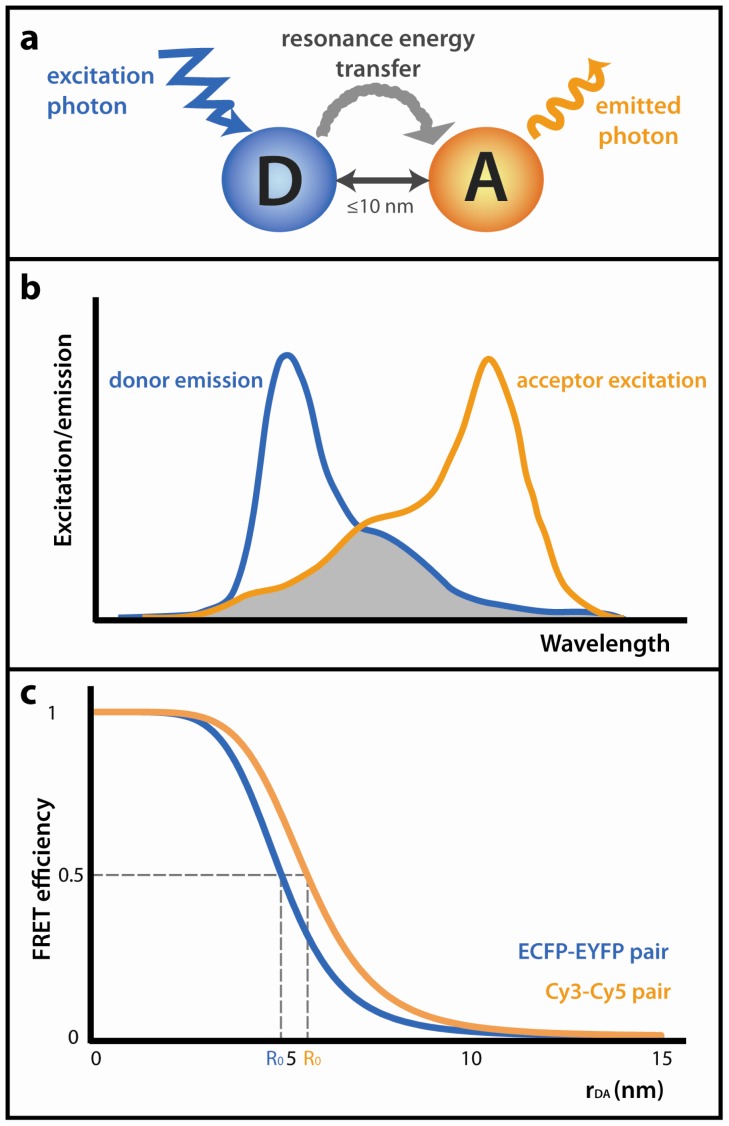
Förster resonance energy transfer (FRET) fundamentals: (**a**) Schematic representation of FRET. Excited donor (D) transfers its energy by a non-radiative process to the nearby acceptor (A), causing it to emit fluorescence. The distance between fluorophores should not exceed 10 nm; (**b**) The donor emission peak must overlap with the acceptor excitation spectrum. The grey area corresponds to the overlap region; (**c**) FRET efficiency as a function of the distance between donor and acceptor fluorophores (*r*_DA_). Förster radius (*R*_0_) of the ECFP-EYFP pair is 4.92 nm [11], *R*_0_ for the Cy3–Cy5 pair is 5.6 nm according to the manufacturer (Amersham Biosciences).

Several conditions must be met for FRET to occur (Figure 1). First of all, donor emission spectra must overlap with acceptor excitation spectra (Figure 1b). As the energy is transferred via dipole-dipole interactions, donor and acceptor must be properly oriented (FRET cannot occur if they are perpendicular to each other). Finally, as FRET efficiency (*E*), defined as the fraction of energy transferred to the acceptor, is inversely proportional to the sixth power of distance between fluorophores, FRET works only at short distances. On average, FRET partners must be less than 10 nm apart for FRET to occur and the dependence of energy transfer on the distance between fluorophores is described in Equation 1:

(1) $$E=\frac{R_{0}^{6}}{R_{0}^{6}+r_{DA}^{6}}$$

where *r*_DA_ means the distance between donor and acceptor molecules and *R*_0_ stands for Förster radius. Förster radius is a parameter unique for each donor-acceptor pair and is defined as a distance at which 50% of energy is transferred from donor to acceptor (Figure 1c). *R*_0_ depends on many optical properties of the FRET pair, e.g., fluorescence quantum yield of the donor, spectral overlap integral of the fluorophores, refractive index and angular dispositions of the FRET pair dipole moments [5].

The best of currently available super-resolution microscopy techniques (reviewed e.g. in [6]) achieve spatial resolution well under the diffraction limit and are able to assess the colocalization of fluorophores on the scale of tens of nanometers [7]. However, even if FRET microscopy does not increase the diffraction-limited resolution of the imaging system, it inherently provides information about fluorophore distances below 10 nm, which is the relevant scale for most biological interactions. See reference [8] for extensive discussion of the merits and drawbacks of FRET microscopy in comparison with super-resolution microscopy techniques.

The most direct approach to measuring FRET is to determine the acceptor emission after donor excitation, which can be easily achieved by using three filter sets with donor/donor, acceptor/acceptor and donor/acceptor excitation/emission wavelengths. However, limitations of this straightforward technique, so-called sensitized emission FRET, are the crosstalk between the emission channels and the direct excitation of the acceptor by the donor excitation wavelength [9]. The need of extensive control measurements makes the technique rather inconvenient for use and other methods are usually utilized to determine FRET in biological samples (reviewed e.g. in [5,10]). For the purpose of this review, we will describe in more detail the FRET applications that are primarily used in the nucleic acid and, more specifically, pre-mRNA splicing field.

### 1.1. Acceptor Photobleaching FRET (AB-FRET)

**Figure 2:**
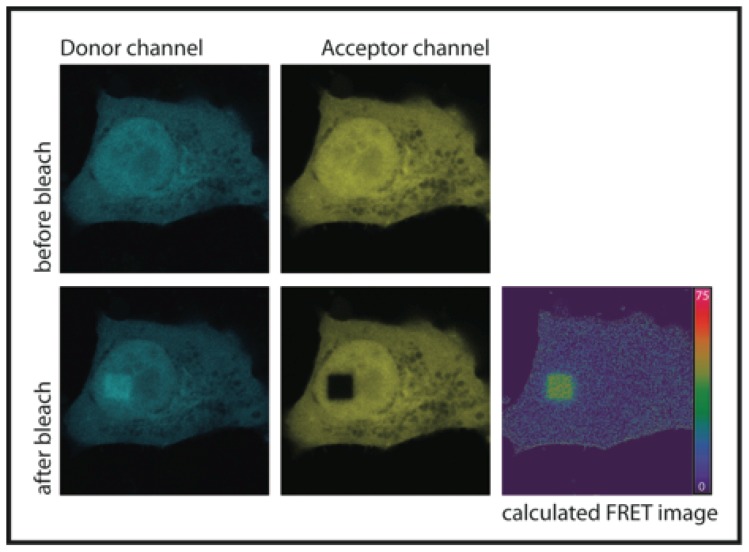
An example of AB-FRET microscopy images of the CFP-YFP fusion protein chimera. Images were acquired with a laser scanning confocal microscope. FRET efficiency was calculated according to Equation 2 and is presented as a false-colored intensity image.

FRET can be measured by monitoring the donor fluorescence quenching by the acceptor. If FRET occurs, the donor emission decreases in the presence of the acceptor. However, for complex biological samples it can be inconvenient to compare fluorescence intensity levels in two separate samples of the donor-only control and donor with acceptor mixture. Even subtle differences between samples, such as different levels of fluorescent protein expression, can bring errors into the measurement. The problem is solved by destroying the acceptor fluorophore by illuminating a region in the sample with a high-power light source (so called photobleaching) and measuring donor fluorescence before and after the photodestruction of the acceptor (Figure 2). The acceptor photobleaching method can be used either with a confocal microscope or with a standard wide-field instrument equipped with lasers, it is relatively easy to implement and no special detectors are needed.

FRET efficiency can be easily calculated from donor fluorescence intensities before (*D*_pre_) and after (*D*_post_) complete destruction of the acceptor (Equation 2)

(2) $$E=\frac{D_{post}-D_{pre}}{D_{post}}$$

To properly determine the FRET efficiency, all acceptor molecules in the region of interest must be successfully photobleached. Incomplete destruction of the acceptor leads to errors in the calculated efficiency [12]. It is also necessary not to affect donor fluorescence during acceptor photobleaching. This can be accomplished by careful choice of the mounting media with an optimal antifade reagent and selection of the wavelength of the photobleaching far away from the donor excitation spectrum [13]. A particular experimental approach should always be tested for the presence of artifacts induced by the photobleaching. Fluorescent proteins can change their emission properties when exposed to specific light wavelengths or thermal activation [14]. This can result in diverse effects, such as reversible photoblinking [15] or photoconversion into a different emission wavelength, which was discussed extensively for the CFP-YFP FRET pair [16–20].

It should be noted that the major strength of AB-FRET lies in measuring FRET efficiency in fixed samples. In live cells, diffusion of unbleached molecules into the bleached area can significantly affect the FRET detection. In addition, the possible phototoxicity of high-intensity laser to live cells must be taken into consideration when shorter (<450 nm) wavelength is used for acceptor photobleaching. Generally, an intensity pulse of several J/m^2^ is required for effective bleaching, which are doses that could induce DNA damage. Therefore it is essential to control for cytotoxicity when performing photobleaching experiments e.g., by observing cell morphology changes after the irradiation or controlling cell passage through mitosis.

### 1.2. Fluorescence Lifetime Imaging Microscopy (FLIM)

**Figure 3:**
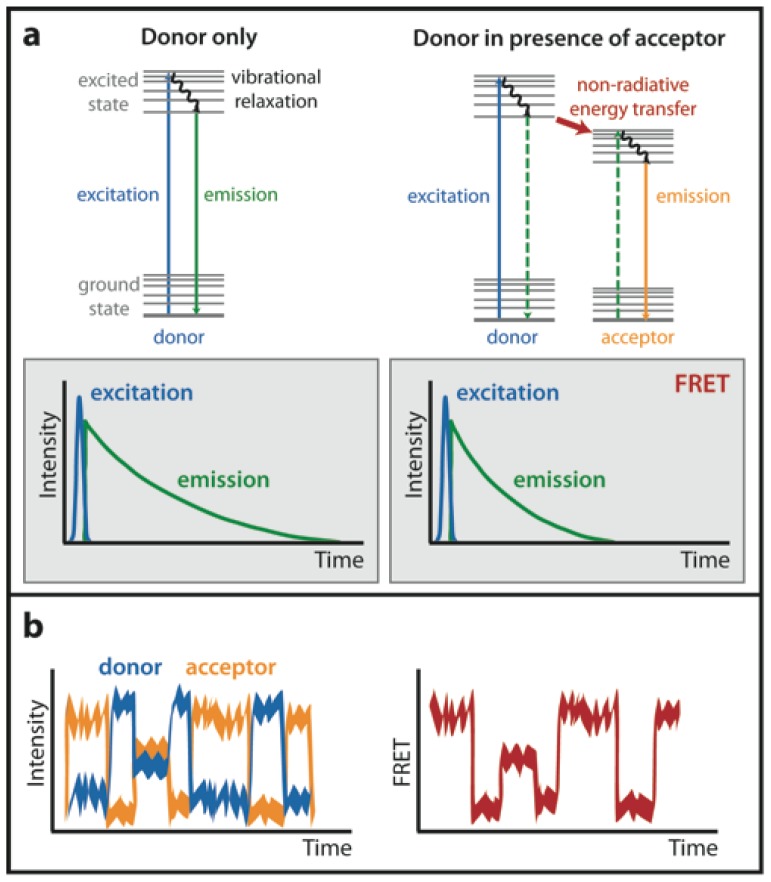
(**a**) Fluorescence lifetime imaging. As is illustrated by Jablonski diagrams, if a fluorophore (donor only) is exposed to the excitation light source, it starts to occupy higher electronic states, and energy is eventually released via vibrational relaxation and emission of fluorescence. The average time the fluorophore spends in the excited state corresponds to its fluorescence lifetime. However, in the presence of a suitable acceptor, the donor can transfer its energy to the acceptor fluorophore non-radiatively and thus return to the ground state. This depopulation of excited state causes shortening of the donor fluorescence lifetime. Model examples of fluorescence lifetime imaging curves are shown in grey boxes. During FLIM measurement in the time domain, the fluorophore is excited by a short pulse and its fluorescence emission is measured over time. If FRET occurs, the fluorescence lifetime decreases; (**b**) Model example of single-molecule FRET. Fluorescence emission after donor excitation is monitored separately in donor and acceptor channels, one molecule at a time. The resulting FRET efficiency is estimated from the D/A fluorescence ratio.

FLIM is a method that measures the decay kinetics of a donor fluorophore excited state. Fluorescence lifetime corresponds to the average time a molecule spends in the excited state before returning to the ground state. The lifetime value depends on the type of the molecule, its conformation and its interactions with the environment [21]. When FRET occurs, a subset of donor molecules transfers energy to acceptors, causing depopulation of the excited state (Figure 3a), and fluorescence lifetime thus decreases. FRET efficiency is proportional to this decrease (Equation 3).

(3) $$E=1-\frac{\tau_{i}}{\tau_{D}}$$

where *τ*_i_ corresponds to the average lifetime detected at position *i* and *τ*_D_ is the lifetime of the donor in the absence of the acceptor.

Fluorescence lifetime of the donor can be measured either in the time domain or in the frequency domain. Frequency domain FLIM monitors the phase shift and demodulation of the fluorescence emission and requires a gain-modulated detector and sinusoidally modulated excitation light. It is used mainly with wide-field microscopes [10]. Time domain FLIM measures the decay curve of emitting fluorophore population after a very short excitation pulse (Figure 3a). It also requires special detectors based on time-correlated photon counting [22] and it is often used in combination with laser-scanning confocal microscopy.

FRET-FLIM is nondestructive and can be conveniently and repeatedly used on live cells, thus providing information about both when and where in the cell the interaction takes place. Fluorescence lifetime is independent of fluorophore concentration and fluorescence intensity [23]. FLIM-FRET is thus very robust to variations in the excitation intensity, microscope geometry and slight changes in donor fluorescence intensity owing to photobleaching [24]. However, FLIM data analysis is technically challenging as curve-fitting algorithms are used for extracting fluorescence lifetimes from the measured datasets and photophysics understanding is needed for data interpretation [25].

### 1.4. Single-Molecule FRET (smFRET)

The sensitivity of FRET measurement can be extended to the single-molecule level (Figure 3b) [26]. This is most commonly achieved by tethering single molecules to a surface such as quartz slide and imaging them by total internal reflection (TIRF) microscopy. smFRET can also be measured in diluted solution of the macromolecules of interest by confocal microscopy [27]. smFRET studies require bright fluorophores with stable emission not influenced by photobleaching or photoblinking. Small organic molecules, such as cyanine dyes, which can be easily covalently attached to biological macromolecules, serve well to this purpose [28]. Extending FRET measurements to the single-molecule level allows observing conformational changes of one molecule at a time, and thus the whole conformational ensemble of a given molecule can be determined. smFRET enables tracking all the conformations on the way from reactants to products, which is advantageous for determining the mechanisms of biological reactions [29]. However, it must be pointed out that smFRET works very well in *in vitro* settings but its application to live cells or complex biological specimens is rather challenging and must be combined with single-particle tracking [30].

## 2. Detection of Nucleic Acid-Protein Interactions *in situ*

### 2.1. Unspecific Labeling of Nucleic Acids

**Figure 4:**
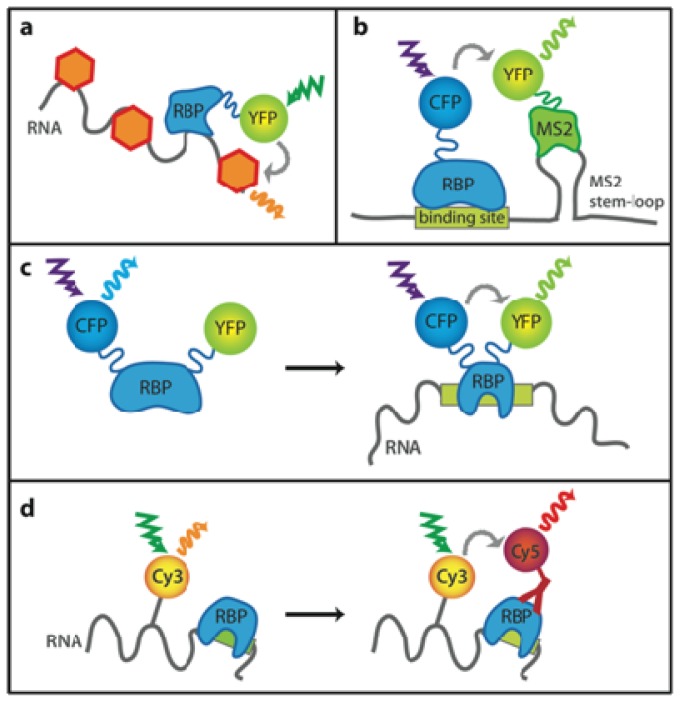
(**a**) Unspecific labeling of RNA by SytoxOrange. When the RNA-binding protein (RBP) tagged with yellow fluorescent protein (YFP) binds to RNA, FRET between YFP and SytoxOrange occurs and can be detected by FLIM; (**b**) RNA-binding mediated FRET technique. RNA with a putative binding site for RBP of interest and MS2 stem loop in close proximity is transcribed from the expression vector. MS2 coat protein tagged with YFP and CFP-tagged RBP are expressed in cells. If RBP binds to the putative binding site, fluorescent protein tags are positioned close to each other and FRET occurs; (**c**) RNA-binding FRET sensor. RBP that changes its conformation after binding to RNA of interest is tagged with a fluorescent donor and acceptor pair. When RBP is bound to RNA, the tags come close enough to each other to enable FRET; (**d**) Direct detection of FRET between RNA and protein. RNA of interest is Cy3-labeled by *in vitro* methods and then injected to cells. RBP of interest is stained with Cy5-labeled specific antibody. FRET between Cy3 and Cy5 is measured afterwards.

FRET can be used for testing whether a protein binds to nucleic acid within the natural cellular environment. Nucleic acids are non-specifically labeled with a fluorescent dye and the protein of interest is tagged with a fluorescent protein. SytoxOrange, a fluorescent molecule that intercalates into DNA and RNA, was used for monitoring interactions between GFP-tagged histone H2B and DNA by FLIM (Figure 4a). DNA-specific labeling was achieved by RNaseA treatment prior to the staining [31]. Interactions of splicing repressors PTB and Raver1 with RNA were monitored by an analogous approach. Cells were treated with DNase prior to SytoxOrange staining and thus only RNA was fluorescently labeled [32]. Yellow spectral variants of fluorescent protein (EYFP and Venus) were used as tags in this FLIM study to provide better spectral overlap between the donor and acceptor spectra [33]. The SytoxOrange technique was also used for monitoring interactions of SR protein family members with nucleic acids in HeLa cells [34] or for verifying carboxyltransferase binding to RNA in *E. coli*[35].

### 2.2. Monitoring Sequence-Specific Binding of Proteins

However, unspecific labeling of a whole population of nucleic acids is not always sufficient. To answer the question whether the protein of interest binds to a specific RNA sequence *in vivo*, Huranova *et al.* developed a method called RNA-binding mediated FRET (Figure 4b) [36]. A putative binding sequence was cloned next to the bacteriophage MS2 binding site and the chimeric RNA expressed in cells together with YFP-tagged MS2 protein and CFP-tagged hnRNP H. The MS2 coat protein binds strongly to its RNA cognate sequence. When a CFP-tagged protein binds the RNA sequence next to the MS2 binding site, it comes into close proximity to the YFP-tagged MS2 protein and FRET occurs.

If a protein changes its conformation upon binding to its nucleic acid target, it can be utilized as a FRET-based sensor (Figure 4c). Endoh *et al.* prepared such sensor from the HIV-1 Rev-peptide cloned between CFP and YFP [37]. When the Rev-peptide interacted with its target Rev response element (RRE) RNA, it changed its tertiary structure. CFP and YFP fluorophores came closer together, and this resulted in FRET. The authors further utilized the method to detect transcription of non-coding RNA containing the RRE element in HeLa cells [38].

FRET can also be observed directly between a nucleic acid molecule and a protein (Figure 4d). The RNA of interest is fluorescently labeled *in vitro* and transfected or microinjected into cells. Interactions between hamster beta adrenergic receptor RNA and mRNA degradation factors, decapping proteins Hedls and Dcp1a, were detected in such a way [39]. Cy3-labeled *in vitro* transcribed mRNA stabilized by 2′ F-dUTP was introduced into a hamster cancer-derived cell line. Endogenous mRNA-binding proteins were labeled via immunostaining with Cy5-conjugated antibodies. The authors were able to distinguish between the mere colocalization and genuine interaction.

## 3. Spliceosome, A Case Study of RNA Interactions Investigated by FRET

**Figure 5:**
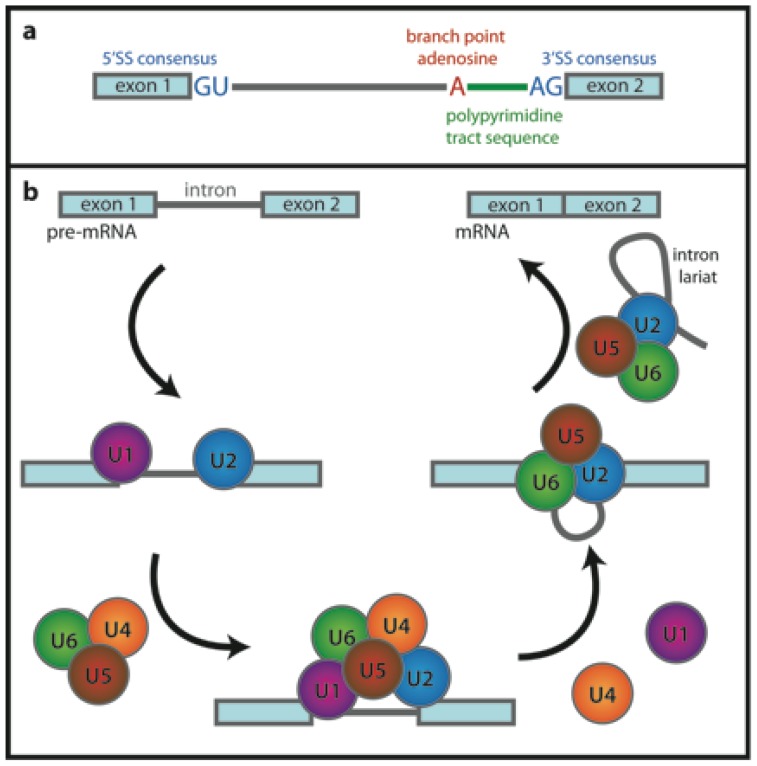
(**a**) Conserved sequence elements of metazoan pre-mRNAs. GU and AG consensus dinucleotides mark intron boundaries. There is a conserved adenosine residue, which serves as a branch point during formation of the intron lariat, and a pyrimidine-rich sequence (polypyrimidine tract) positioned between the branch point and conserved AG at the 3′SS; (**b**) Simplified scheme of the spliceosome assembly.

Primary transcripts of eukaryotic cells contain intervening sequences (introns), which must be spliced out before translation. This process, called pre-mRNA splicing, is carried out by a multi-megadalton complex—the spliceosome [40], which assembles step-by-step on the pre-mRNA molecule [41]. Pre-mRNA splicing is a process of two subsequent trans-esterification reactions. First, the 2′-hydroxyl group of branch point adenosine (Figure 5a) attacks the phosphodiester bond at the 5′ splice site (SS). This generates an intron lariat attached to the exon 2 and frees the 3′-OH of the exon 1, which subsequently attacks the phosphodiester bond at the 3′SS. Second trans-esterification occurs, the exons are joined together and the intron lariat is released.

Pre-mRNA splicing is catalyzed by the spliceosomal complex, which consists of five core small nuclear ribonucleoprotein particles (U1, U2, U4, U5 and U6 snRNPs) and additional proteins (Figure 5b). First, the U1 snRNP binds to pre-mRNA at the 5′SS followed by U2 auxiliary factor and U2 snRNP binding to the 3′SS. The pre-assembled U4/U6·U5 tri-snRNP then joins the complex and after dissociation of U1 and U4 and several conformational rearrangements the spliceosome is activated and catalyzes two trans-esterification steps of the splicing reaction. After that, mature mRNA is released and U2, U5 and U6 snRNPs dissociate from the intron lariat and are recycled. The spliceosome assembly pathway has been nicely reviewed in recent literature, e.g., [42,43].

### 3.1. RNA Conformational Dynamics

Pre-mRNA and snRNAs in the spliceosome are not rigid during splicing. Until recently we had only indirect evidence about their conformational changes. However, smFRET proved to be helpful in monitoring these changes [44]. Abelson and colleagues prepared a splicing reporter derived from *S. cerevisiae* Ubc4 pre-mRNA and introduced fluorescent labels (Cy3 and Cy5) into two adjacent exons. FRET between Cy3 and Cy5 was monitored over time, revealing that the reporter pre-mRNA molecule is highly dynamic and undergoes transitions between several folding states. These results indicate that the splice sites can be positioned close to each other even in the absence of splicing extract. smFRET measurement in the presence of yeast extract capable of *in vitro* splicing showed several reversible conformational states of pre-mRNA in a complex with spliceosomal proteins. This suggests that spliceosome works under near-equilibrium conditions rather than just following a simple unidirectional reaction pathway.

### 3.2. Early Complex Assembly

**Figure 6:**
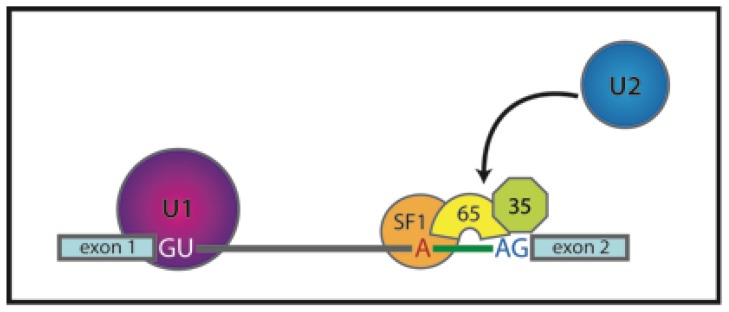
Schematic representation of the early stage of spliceosome assembly. 5′SS is bound by U1 snRNP. U2AF35 binds 3′SS, U2AF65 interacts with the polypyrimidine tract and stabilizes interaction of SF1 with the branch point. The U2AF heterodimer recruits U2 snRNP to the 3′SS.

During the early stage of spliceosome assembly, the 3′SS consensus sequence is recognized by the U2 snRNP auxiliary factor (U2AF). U2AF recruits U2 snRNP to pre-mRNA and leaves the complex during later stages of spliceosome assembly. U2AF is a heterodimer consisting of 65 kDa and 35 kDa subunits (U2AF65 and U2AF35). U2AF65 stabilizes interaction of the U2 snRNP with the branch point while U2AF35 binds the conserved AG dinucleotide at the 3′SS (Figure 6) [45,46].

U2AF extensively interacts with splicing factors, but where and when these interactions occur within cells was not known. FRET studies revealed that the subunits of U2AF directly interact with each other even in the absence of transcription, suggesting that the U2AF heterodimer is preassembled before binding to pre-mRNA and stored in distinct foci called nuclear speckles [47]. U2AF65 also associates with splicing factor 1 (SF1) enhancing its binding to the pre-mRNA branch point sequence. SF1 is later replaced with U2 snRNP proteins. AB-FRET and FLIM measurements showed that SF1 interacts with both subunits of U2AF even in the absence of pre-mRNA and these extraspliceosomal complexes also localize in nuclear speckles [48].

### 3.3. SR-Protein Interactions during Intron Recognition

Intron recognition is not based solely on the consensus sequences at intron 5′ and 3′ ends. Short RNA motifs around splice sites help to navigate and regulate the splicing machinery. These RNA elements are bound by regulatory proteins that consequently recruit other parts of the carefully orchestrated splicing machinery [49]. One of the most important families of these protein regulators are highly conserved serine/arginine rich (SR) proteins. SR proteins bind to splicing enhancer sequences in pre-mRNA and are able to enhance recruitment of snRNPs to the splice sites and thus to promote exon recognition [50].

To directly monitor SR protein interactions with RNA *in situ*, Sapra *et al.* utilized the SytoxOrange labeling in HeLa cells [34]. They used FRET-FLIM to confirm interactions of GFP-tagged SR proteins with RNA. One of the proteins, SRSF2, also bound to DNA and failed to shuttle between the nucleus and the cytoplasm, in contrast to the other investigated SR proteins. To map the interactions of SR proteins with the basal splicing machinery *in situ*, Ellis and co-workers utilized FRET-FLIM [51]. They mainly analyzed complexes of SR proteins with U1 snRNP and U2AF. These interactions occurred in both nuclear speckles and nucleoplasm. The FRET signal in nuclear speckles increased after transcription inhibition, whereas FRET efficiency measured in the nucleoplasm decreased significantly. This is in agreement with the view that posttranscriptional splicing occurs in nuclear speckles [52].

### 3.4. Formation of the U4/U6·U5 Tri-snRNP

After binding of U1 and U2 snRNPs to appropriate splice sites, the pre-assembled U4/U6·U5 tri-snRNP joins the complex. Assembly of the active U4/U6·U5 tri-snRNP is a step-wise process and the first step involves U4/U6 di-snRNP assembly during which U4 and U6 base-pair with each other and U4/U6 specific proteins attach. U4/U6 formation is initiated by binding of the 15.5K protein to the U4 stem-loop. This interaction is needed for other proteins to join the di-snRNP [53]. Structural changes of U4 snRNA in the presence of 15.5K were monitored *in vitro* using smFRET. It was shown that the specific structural motif, kink-turn, on U4 snRNA was induced after 15.5K protein binding [54].

Further steps of U4/U6 snRNP assembly are promoted by SART3 and LSm proteins. Here, AB-FRET became extremely useful because it allowed detection of different snRNP assembly intermediates inside the cell nucleus. SART3·U6 snRNP was found exclusively in the nucleoplasm, whereas SART3 associated with U4/U6 di-snRNP preferentially localized to the nuclear compartment called the Cajal body. These data showed that the snRNP assembly process is compartmentalized and Cajal bodies are sites of di-snRNP assembly [55]. Recently, a molecular bridge that anchors the U4/U6 snRNP to the Cajal body was identified by AB-FRET. Novotny and colleagues showed that SART3 interacts with coilin, a structural protein of Cajal bodies [56]. The U5 snRNP attachment to the di-snRNP and formation of the tri-snRNP also occurs in Cajal bodies [57].

### 3.5. FRET Helps to Solve the Catalytic core Structure and Dynamics

**Figure 7:**
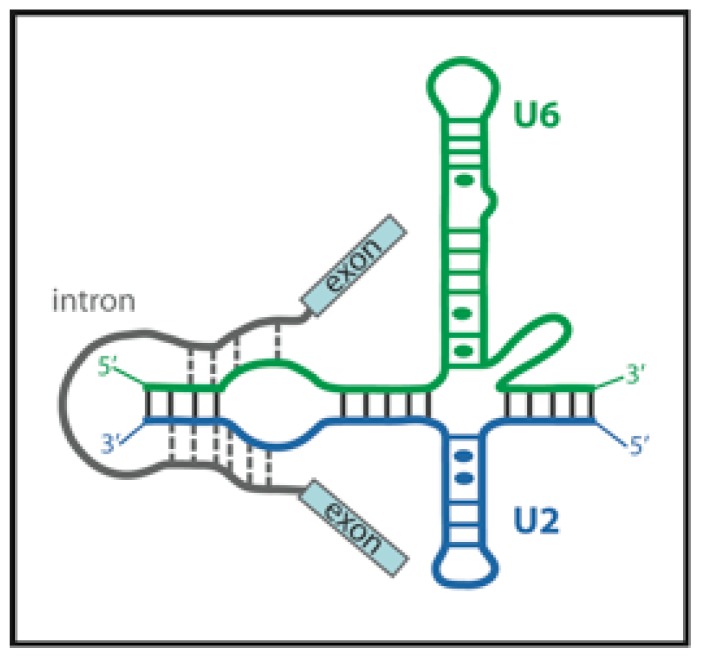
Schematic representation of the spliceosomal core. Secondary structure model of the spliceosomal snRNAs U2–U6 from *Saccharomyces cerevisiae* with an intron bound. Watson-Crick base-pairs are depicted as lines, non-Watson-Crick pairs as circles. Adapted from [58].

After U4/U6·U5 tri-snRNP joining, the spliceosome undergoes extensive rearrangements. U1 and U4 snRNPs leave and the active spliceosome containing U2, U5 and U6 snRNAs forms and catalyzes both splicing steps. The available data suggest that the catalytic core of the spliceosome is formed by base-paired U2 and U6 snRNAs (Figure 7). The conformational changes of the protein-free U2–U6 snRNA complex were monitored by smFRET. These experiments revealed that the complex can adopt at least three distinct conformational states in equilibrium [58]. One of the inter-conformational transitions is strongly magnesium-dependent. The observed conformational states likely correspond to rearrangements of the spliceosomal core during the intron removal catalysis.

The position of divalent ions that are crucial for spliceosome catalytic activity was monitored by Yuan *et al.* They applied an elegant luminescence resonance energy transfer (LRET) approach to map the positions of magnesium ion binding sites [59]. They used terbium Tb (III) ion as a donor of energy to be transferred to Cy3 acceptor covalently attached to a snRNA molecule. Terbium is a lanthanide metal and when in ionic state, it has chemical and physical properties very similar to divalent alkaline earths metals, such as magnesium. The authors exchanged magnesium in the U2–U6 complex for luminescent terbium ions and monitored transfer of energy to the Cy3 acceptor by FLIM *in vitro*. They were able to detect three distinct metal ion binding sites; two of them pH dependent. The authors speculate that such pH-dependent ion binding may serve as a regulatory mechanism for modulating splicing activity. However, the mechanism of such pH changes in the cellular microenvironment is still unclear.

### 3.6. Probing Alternative Splicing with FRET

**Figure 8:**
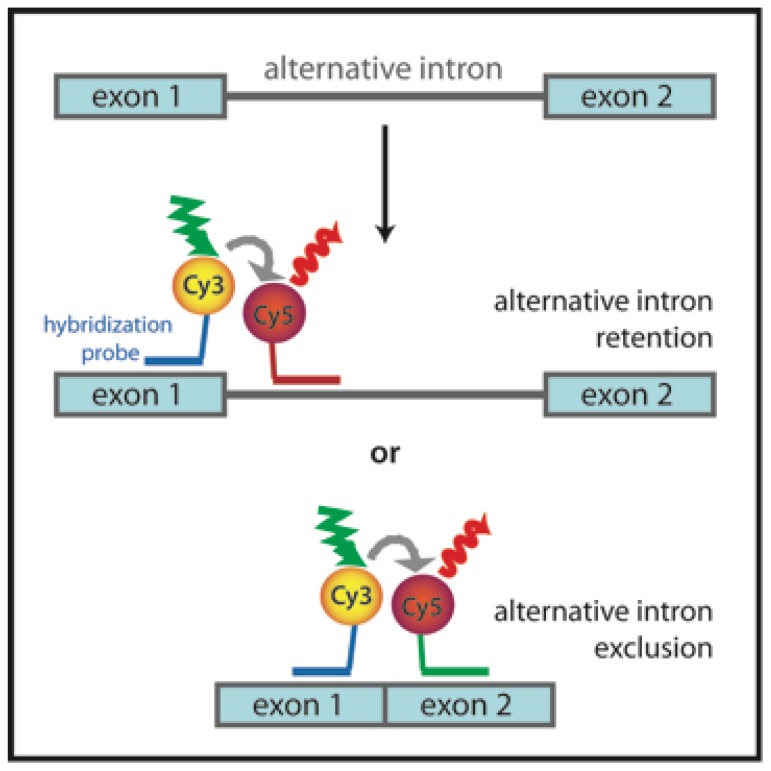
Alternative splicing detection via *in situ* hybridization according to [61]. Cy3-labeled hybridization probe complementary to exon 1 is used together with either exon 2 or alternative intron complementary Cy5-labeled probe. FRET occurs when probes are bound to mRNA close to each other.

Alternative splicing is an elegant way of complex organisms to boost the coding potential of their genomes and produce more than one protein from a single gene. Bioinformatics combined with genomic approaches revealed that approx. 95% of human genes are alternatively spliced [60]. To analyze alternative splicing directly in cells, several FRET-based approaches were developed. Blanco *et al.* used a fluorescence *in situ* hybridization (FISH) approach to detect various alternatively spliced transcripts *in situ* in fixed cells [61]. They were able to detect isoforms of pre-mRNA of lymphocyte antigen 6 gene in HeLa cells and discriminate between intron removal and retention. Hybridization probes complementary to exon sequences flanking splice sites end-labeled with Cy3 or Cy5 were used. If an intron was spliced out of pre-mRNA, the probes came close enough to each other to allow detection of FRET (Figure 8). The method seems to be sensitive enough to allow characterization of the targeting and processing of alternatively spliced transcripts *in situ*.

## 4. Conclusion and Outlook

Despite 65 years since the FRET phenomenon was first described by Theodor Förster, FRET microscopy still represents a powerful approach that helps to answer crucial questions of cell biology. FRET has been helping to understand compartmentalization of individual steps of spliceosome formation in the cell nucleus and to reveal the dynamics of splicing reaction and structure of the catalytic core. With increasing microscope speed and sensitivity, single-molecule techniques will become widely used and FRET will become a method of choice for describing the molecular mechanisms of different aspects of nucleic acid metabolism with a single-molecule resolution.
